# Cat-Scratch Disease Mimicking Neoplastic Etiology in a Complex Clinical Presentation: A Case Report

**DOI:** 10.7759/cureus.66840

**Published:** 2024-08-14

**Authors:** Evan Smith, Rob Lawless, Andrew Hoellein

**Affiliations:** 1 Internal Medicine, University of Kentucky College of Medicine, Lexington, USA

**Keywords:** pcr detection of b. henselae, concomitant human immunodeficiency virus (hiv) infection, bartonella henselae, massive cervical lymphadenopathy, hiv lymphoma, cat scratch

## Abstract

Cat-scratch disease (CSD), caused by *Bartonella henselae* (*B. henselae*), typically presents with regional lymphadenopathy following a cat scratch or bite. We report a case of a 50-year-old man with a complex medical history including HIV, Crohn's disease, coronary artery disease, and bipolar disorder, who presented with progressively enlarging cervical lymphadenopathy associated with fever, night sweats, and myalgias. Initial evaluation suggested a neoplastic etiology, prompting extensive laboratory investigations and imaging. However, subsequent history prompted serological testing and markedly elevated *Bartonella* antibody titers, leading to a clinical diagnosis of CSD. Empirical doxycycline therapy was initiated, resulting in the complete resolution of symptoms. This case underscores the importance of considering CSD in the differential diagnosis of lymphadenopathy, particularly in people living with HIV regardless of immunocompetency, and highlights the challenges of diagnosis and management in complex patients.

## Introduction

Cat-scratch disease (CSD) is a bacterial infection commonly caused by the gram-negative bacterial species *B. henselae*. The disease may spread to humans when an infected cat bites or scratches the skin hard enough to puncture the outer layer of the skin, although some cases have reportedly been transmitted from cats licking open wound sites [1]. The disease most commonly presents three to 14 days after initial exposure, and symptoms often include inflammation around the infected site, fever, regional lymphadenopathy, and rare instances of endocarditis and encephalitis [1,2,3]. *B. henselae* causes CSD by inducing regional lymph nodes to drain the area of initial exposure, after which a granulomatous response will occur [2]. The affected lymph nodes will then become swollen, enlarged, and tender, with the axillary lymph nodes being involved in most cases [1,2]. CSD can also lead to chronic lymphadenopathy, which can affect nodes outside of the area of exposure [2]. In immunocompetent patients, CSD typically causes a mild illness. In immunocompromised patients, *Bartonella* can cause opportunistic infections such as bacillary angiomatosis, which can present with red/brown papules that resemble those found in Kaposi sarcoma [3]. Diagnosis of CSD can be difficult to achieve due to the difficulty of culturing *B. henselae*; therefore, exposure to cats or a history of a cat bite or scratch is often necessary for diagnosis [2]. Lymph node biopsy, although not indicated routinely, can be used for diagnosis in difficult cases [2].

## Case presentation

A 50-year-old man with a past medical history of well-managed HIV, Crohn's disease, coronary artery disease, and bipolar disorder presented for the management of chronic disease, including evaluation of a newly noticed enlarged lymph node. He reported that one week prior, he had discovered a swollen, firm, non-tender, and mobile lymph node located just below his right jawline. The lymph node had progressively grown over this time. He also noted that over the past few days, he had had a low-grade fever (~100°F). He denied any other symptoms or recent sick contacts and demonstrated a negative COVID-19 test.

**Figure 1:**
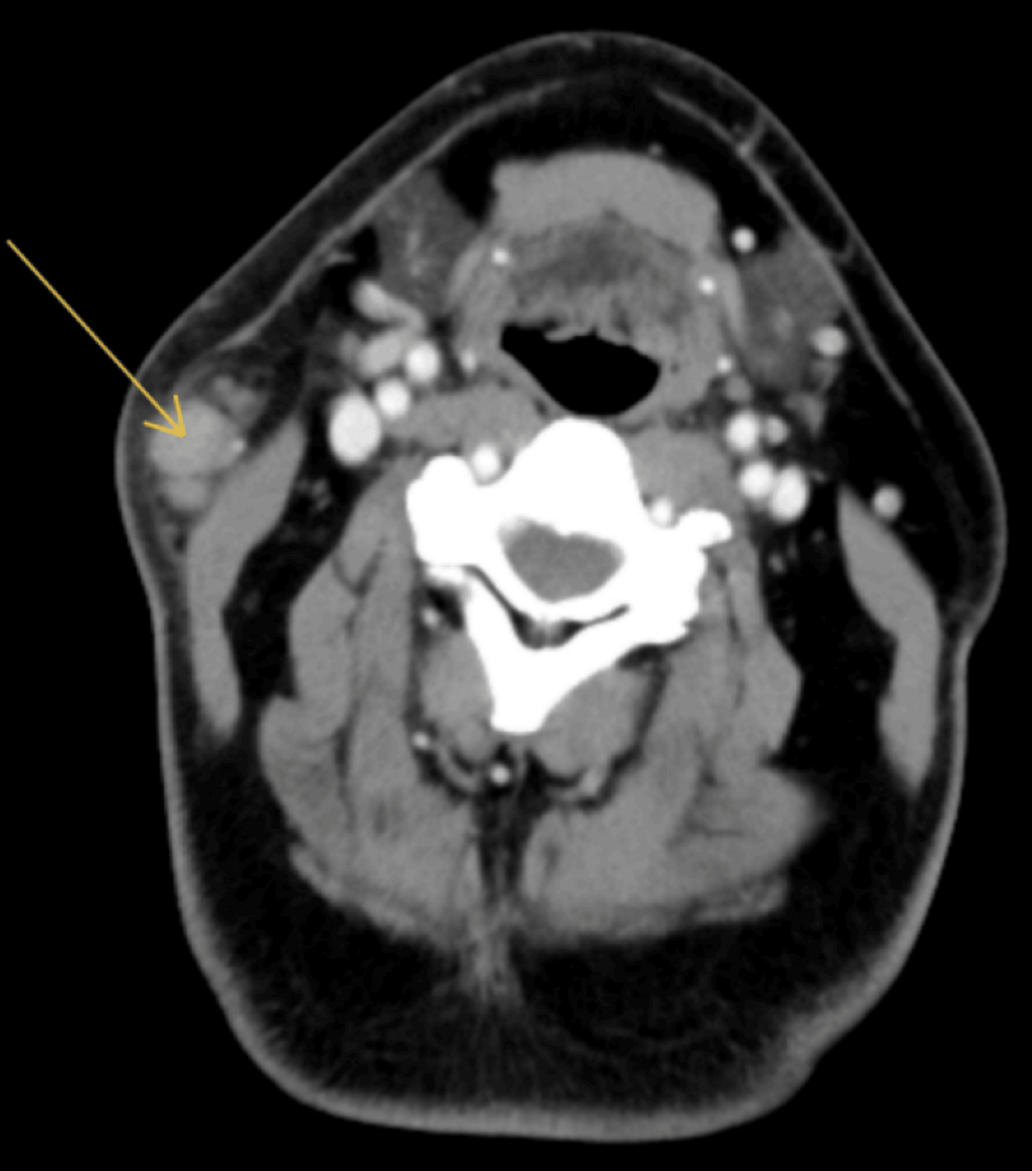
Axial CT denoting enlarged cervical lymph node with surrounding soft tissue stranding

**Figure 2:**
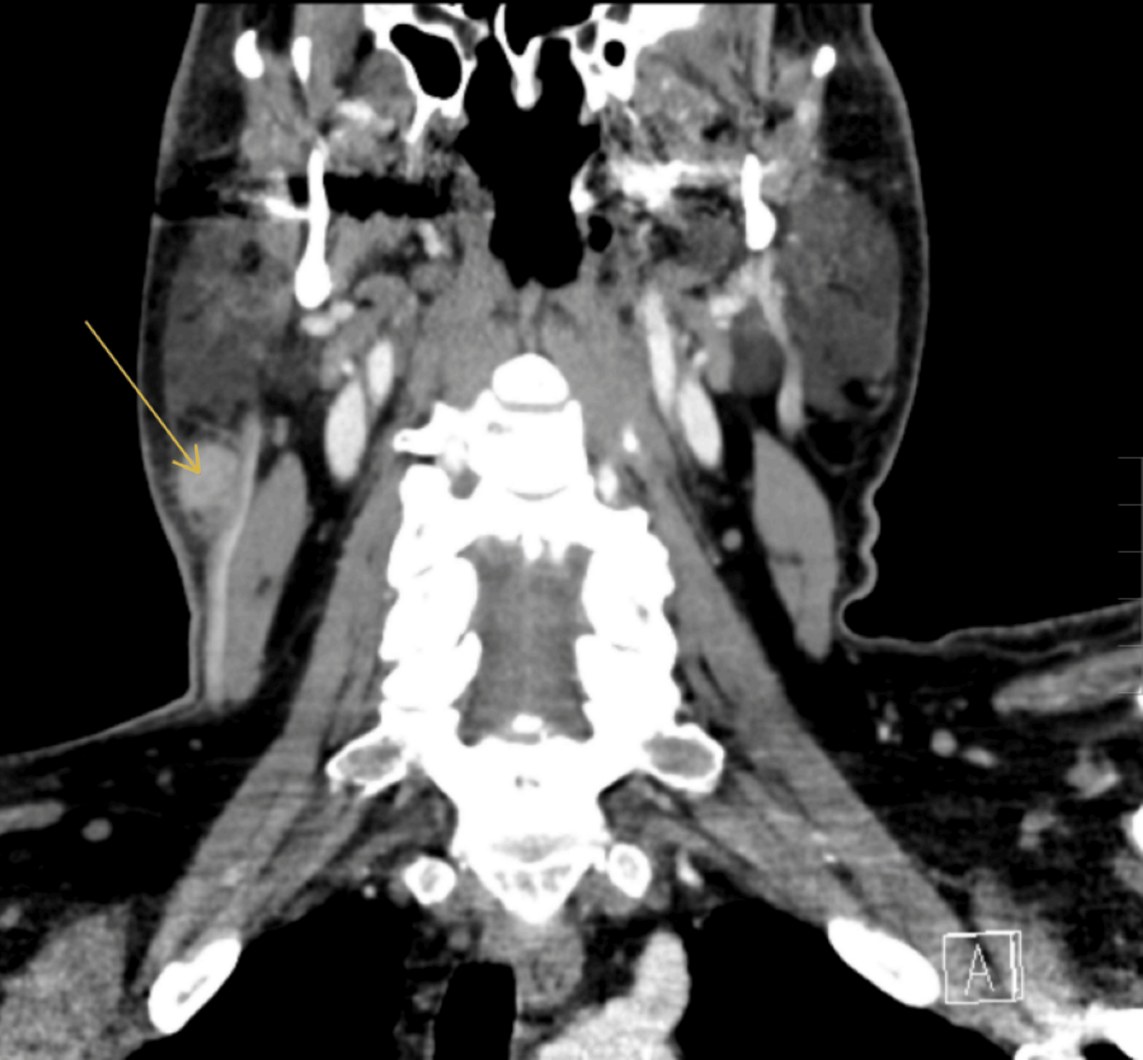
Coronal CT denoting prominent cluster of cervical lymph node enlargement

Upon follow-up one week later, he reported high fevers (~103.5°F), night sweats, nausea, vomiting, myalgia, and persistent adenopathy. A neoplastic etiology was suspected, and further investigations were undertaken. A neck CT with IV contrast (Figures 1, 2) was ordered, which revealed a cluster of large (1.3 x 1.4 cm on the orthogonal axis) enhancing soft tissue nodules immediately inferior to the right parotid tail. Right-sided intermediately sized (1.4 x 0.7 cm) cervical lymph nodes were also noted nearby. Extensive laboratory assessments were ordered, including complete blood cell count with a differential, comprehensive metabolic panel, *Neisseria gonorrhea *polymerase chain reaction (PCR), *Chlamydia trachomatis* PCR, lymphocyte subset enumeration, HIV quantitative viral load, rapid plasma reagent, and blood cultures. Fine needle aspiration of the submandibular node was sent for pathology and leukemia/lymphoma immunophenotyping by flow cytometry. All studies were normal or non-indicative except for the CBC, which demonstrated an elevated white cell count of 15.59 x 10^3^/uL with both neutrophils and lymphocyte counts elevated. Empiric doxycycline treatment (100 mg twice per day x 10 days) was started.

**Figure 3:**
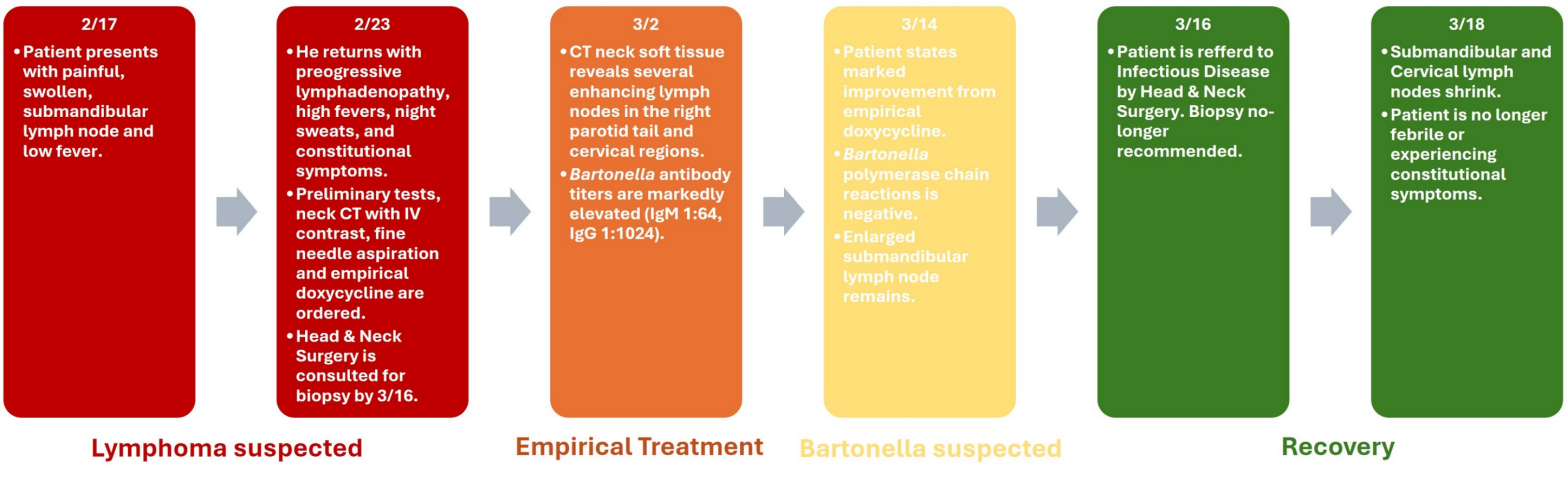
Timeline of disease progression CT: computed tomography; Ig: immunoglobulin; IV: intravenous

These results seemed indicative of an infectious or inflammatory etiology rather than neoplastic. Further testing was obtained one week later, including complement, EBV antibody, mononucleosis, *Bartonella* IgG and IgM, anti-nuclear antibody (ANA), C-reactive protein, sedimentation rate, rheumatoid factor, and blood cultures. *Bartonella* titers showing extreme elevation (IgG >1:1024, IgM 1:640) were the most revelatory. Subsequently, a clinical diagnosis of *Bartonella henselae* infection, also known as cat-scratch disease (CSD), was made. It was later revealed that the patient not only owned a cat but also routinely volunteered at an animal shelter. Exposure to kittens is a well-known and significant risk factor for CSD. Targeted doxycycline treatment was continued while confirmatory testing with *Bartonella* DNA PCR was conducted. By this time, the *Bartonella* PCR was returned negative, although the clinical diagnosis remained. At this point, the patient had been symptom-free for over a year (Figure 3).

## Discussion

CSD, which is caused by the bacteria *B. henselae*, presents three to 14 days after a cat scratch or bite [1,2,3]. Initial symptoms include inflammation and redness near the inoculation site and can progress to regional lymphadenopathy throughout the course of the illness [4]. Rare cases, such as those who are immunocompromised, can proceed to encephalitis or endocarditis [2,3]. Care should be taken to differentiate between neoplastic lymphadenitis and CSD [5]. In many cases, the symptoms of the two etiologies can often present quite similarly. Lymphadenopathy, fever, fatigue, and night sweats are often common features of both conditions. In CSD, axillary lymphadenopathy is most common, occurring in 43% of patients and cervical and submandibular in only 28% of presentations as seen in this case [6]. Although the lymphadenopathy associated with CSD often resolves spontaneously, early diagnosis and intervention are important to avoid dissemination to other organs throughout the body [4]. The clearest way to distinguish between the two conditions is through blood testing, including but not limited to CBC, blood cultures, and *Bartonella* antibody titers.

When the patient presented to the clinic with cervical lymphadenopathy, constitutional symptoms, and immunosuppression via their HIV diagnosis, neoplastic etiology such as lymphoma was initially very high on the differential. The patient’s HIV viral load was measured, and it was within normal range, leading to the conclusion that the patient’s HIV was well under control with subsequent classification of immunocompetent. Immunosuppression is not required for a diagnosis of lymphoma or any other neoplastic condition, so a CBC with differential, blood cultures, and other testing was done to analyze pertinent values to determine the etiology of the symptoms the patient was experiencing. Fine needle aspiration of the lymph node was also done. When these tests returned and no abnormal values were found, it was clear that this was not a neoplastic condition, and further history was obtained to determine if any infectious or inflammatory etiology was the cause of the symptoms. When the patient relayed that they not only owned cats, but also volunteered at an animal shelter, CSD via* Bartonella *infection immediately rose to the top of the differential. *Bartonella *titers were extremely elevated, leading to the diagnosis of CSD.

Although CSD and lymphoma have very different etiologies, they can present very similarly in patients. The presence of cervical or axillary lymphadenopathy, constitutional symptoms, malaise, nausea, and vomiting can be symptoms of both conditions, and CSD can often be omitted if a proper history is not obtained detailing potential animal exposures.

## Conclusions

CSD, caused by *B. henselae* infection, most commonly occurs after being scratched or bitten by a cat. Symptoms include inflammation and erythema near the sight of inoculation, followed by fever, malaise, and regional lymphadenopathy two to three weeks after infection. If laboratory results are not indicative of a neoplastic etiology, *B. henselae *infection should be excluded even in rare instances of cervical lymphadenopathy. Radiologic imaging, *Bartonella* antibody titers, and PCR are indicated for the diagnosis of this condition.
